# The Potential for Big Data in Animal Disease Surveillance in Ireland

**DOI:** 10.3389/fvets.2017.00150

**Published:** 2017-10-06

**Authors:** Damien Barrett

**Affiliations:** ^1^Surveillance, Animal by Products and TSE Division, Department of Agriculture Food and the Marine, Celbridge, Ireland

**Keywords:** surveillance, data bases, data mining, syndromic surveillance, production animals

## Introduction

Animal Health Surveillance is the systematic collection, collation, analysis interpretation, and dissemination of animal health and welfare data from defined populations. This process is essentially about gathering intelligence to detect either novel animal health-related events or increases in animal health-related events as early as possible to better inform risk management at all levels within the industry (1). The incursion of an exotic disease, such as Foot and Mouth disease, is probably the most significant event from a national animal health perspective. The early detection of a newly emerging disease or the re-emergence of a disease which was previously eradicated from the state before it becomes widespread in the population is another important objective of a well functioning surveillance system. From a trade perspective, it is hugely important to demonstrate freedom from specific diseases, and this requires objective evidence upon which to base any declarations of freedom. While the presence of endemic diseases will be well established, there is a specific need to monitor the occurrence of endemic disease to identify any spikes in their occurrence and to take appropriate risk mitigation actions. Antimicrobial resistance (AMR) is emerging as one of the most significant public health concerns of the twenty-first century. The use of antimicrobials in livestock has come under increased scrutiny as a result. The monitoring of the use of the levels of antimicrobials in livestock and the emergence of resistance among animal pathogens are two important surveillance priorities in the years ahead. Animal health surveillance is a part of animal disease risk management in that the information garnered should lead to better informed risk mitigation strategies. These risk mitigation strategies must deal with risks that are well established as well as risks which are not yet fully understood, and therein lies the challenge.

## Data—Not all Data are Equal

**Figure 1:**
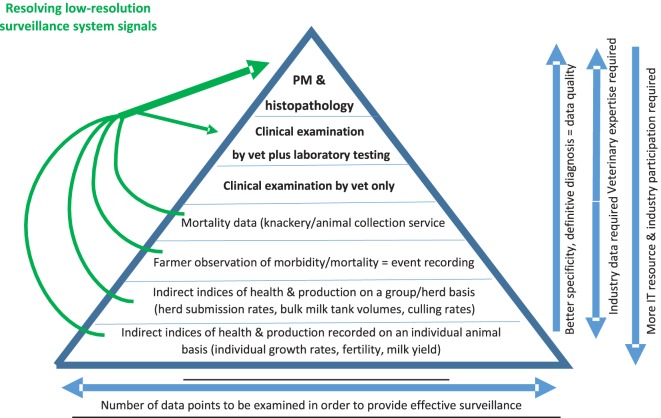
The surveillance pyramid: quality vs width in animal disease surveillance.

Various data can be analyzed for surveillance purposes (Figure 1). However, the value of these sources cannot all be considered equal. Data acquired from postmortem examination from scanning surveillance is of a low volume but has a high resolution due to the detailed level of examination. This is followed in terms of precision by clinical examination by veterinary practitioners, where there is a larger volume of data, but it is considered less accurate than postmortem examination. Trends in mortality data, followed by trends in disease event recording are next in terms of data usefulness. Production data from herds and individual animals contains much more data points, with significant background noise interfering with the information. Data only becomes information when they are processed and these larger data sets need considerable data mining and analysis to make them into information which can be interpreted.

## Current Methods of Gathering Surveillance Information in Ireland

Currently, there is a network of six Regional Veterinary Laboratories in Ireland which gather passive surveillance data on submissions made by farmers on referral by their private veterinary practitioners. Submissions are made up of carcasses for postmortem examination and blood/feces samples for clinical pathology. However, the postmortem data are generally considered the more detailed and hence more valuable. An underutilized aspect of the current system is the soft intelligence created through conversations with private veterinary practitioners is not currently recorded nor analyzed. An analysis of telephone calls to Dutch animal health services in 2011, identified an increase in the prevalence of syndromes associated with the occurrence of Schmallenberg virus (SBV) in the weeks prior to the confirmed emergence of SBV (2). Serosurveys are carried out from time to time to gather information on the prevalence of diseases of particular interest. There are legislative requirements to survey various species annually to verify freedom from various diseases. These sampling frames have been used to carry out surveillance for other infections, such as SBV (3). Bulk milk antibody testing provides another method to get an insight into the health of dairy herds. Such serological testing tends to be of limited value where endemic disease has been established. Individual research projects are carried out from time to time on diseases of particular interest.

The Irish cattle industry is in the unique position of having its production database, which is operated by the Irish Cattle Breeding Federation (ICBF), directly linked to the Department of Agriculture, Food and the Marine’s Animal Identity and Movement database. While the ICBF database’s primary function relates to genetic and production data, these data could be used for animal health monitoring purposes. Similar databases in other countries have been successfully analyzed to find trends in mortality (4).

## Future Opportunities

While there are vast volumes of data, which are not well integrated, integration could facilitate data mining and the use of data analytics to identify animal health-related risk earlier, so that those risks could be managed more efficiently. The costs of dealing with the animal health incidents at herd and national levels are disproportionately greater than the costs of carrying out comprehensive risk assessments using data analytics (5). An effective surveillance system will identify events sooner, and thereby allow corrective/preventive actions to take place sooner, and thereby limit the consequences of that event. However, in many countries, the availability of data and the analysis of those data is currently the limiting step. Data relating to weather, product price, and socioeconomic factors could also be integrated into such an approach. Modern milking systems have the ability to record yields per cow at each milking and such drops in milk yield has the potential to identify animal health at individual animal, herd, and regional levels.

Syndromic surveillance has been used in human public health policy to identify trends in public health. Databases relating to school and workplace absenteeism, General practitioner treatment records, Accident and Emergency treatment records and over the counter medicine sales have provided syndromic data to be analyzed, looking for changes in demand patterns which can be linked to the health-related events in the general population. While there are no comparable data in veterinary medicine, there is potential to get information on diseases which are seen by veterinary practitioners but are not regulated by government. The emergence of AMR is directly related to the use of antimicrobials (6), and therefore, the monitoring of antimicrobial usage at individual farm level is an important element of control of AMR. Syndromic surveillance also offers an opportunity to monitor the actual usage of antimicrobials on farm, rather than sales. There is likely to be greater demand for such information in the future, to better inform the management of AMR.

The Irish dairy industry has embarked on a period of considerable expansion, as set out in Food Harvest 2020 and Foodwise 2025, two policy documents setting a strategic framework for the development of agriculture in Ireland (7, 8). In Ireland, we are fortunate to have large vast quantities of animal health-related data residing in national, veterinary practice and individual farm databases. While the techniques to interrogate these data for animal health risk management purposes have been developed elsewhere (9), their application in an Irish context has been limited to date.

## Author Contributions

The author confirms being the sole contributor of this work and approved it for publication.

## Conflict of Interest Statement

The author declares that the research was conducted in the absence of any commercial or financial relationships that could be construed as a potential conflict of interest.
